# Evaluating and Calibrating Uncertainty Prediction in Regression Tasks

**DOI:** 10.3390/s22155540

**Published:** 2022-07-25

**Authors:** Dan Levi, Liran Gispan, Niv Giladi, Ethan Fetaya

**Affiliations:** 1 General Motors Israel, Herzliya 4672515, Israel; liran.gispan@gm.com (L.G.); giladiniv@gmail.com (N.G.); 2 Faculty of Computer Science, Technion, Haifa 3200003, Israel; 3 Faculty of Engineering, Bar-Ilan University, Ramat Gan 5290002, Israel; ethan.fetaya@biu.ac.il

**Keywords:** regression, prediction uncertainty

## Abstract

Predicting not only the target but also an accurate measure of uncertainty is important for many machine learning applications, and in particular, safety-critical ones. In this work, we study the calibration of uncertainty prediction for regression tasks which often arise in real-world systems. We show that the existing definition for the calibration of regression uncertainty has severe limitations in distinguishing informative from non-informative uncertainty predictions. We propose a new definition that escapes this caveat and an evaluation method using a simple histogram-based approach. Our method clusters examples with similar uncertainty prediction and compares the prediction with the empirical uncertainty on these examples. We also propose a simple, scaling-based calibration method that preforms as well as much more complex ones. We show results on both a synthetic, controlled problem and on the object detection bounding-box regression task using the COCO and KITTI datasets.

## 1. Introduction

Regression problems are common machine learning tasks and in many applications, simply returning the target prediction is not sufficient. In these cases, the learning algorithm also needs to output its confidence in its prediction. For example, when the predictions are used by a self-driving car agent, or any other safety critical decision maker, it needs to take the confidence of these predictions into account. Another example is the commonly used Kalman filter tracking algorithm [1] that requires the variance of the observed object’s location estimation in order to correctly combine past and present information in order to better estimate the current state of the tracked object.

To provide uncertainty estimation, each prediction produced by the machine learning module during inference should be a distribution over the target domain. There are several approaches for achieving this: most common are Bayesian neural networks [2,3], ensembles [4] and directly outputting a parametric distribution [5]. For simplicity, we use the direct approach for producing uncertainty: we transform the network output from a single scalar to a Gaussian distribution by taking the scalar as the mean and adding a branch that predicts the standard deviation (STD) as in [4]. While this is probably the simplest form, it is commonly used in practice, and our analysis is applicable to more complex distributions as well as other approaches.

Given the output distributions over target domains and observed targets, the main question we addressed in this work is how to evaluate the uncertainty estimation of our regressors. For classification, a simple and useful definition is calibration. We say that a classifier is calibrated if, when it predicts some label with probability *p*, it is correct with exactly probability *p*. Recently, it has been shown that modern deep networks are not well calibrated but rather tend to be over confident in their predictions [6]. The same study revealed that, for classification, Platt Scaling [7], a simple scaling of the logits, can achieve well-calibrated confidence estimates.

Defining calibration for regression, where the model outputs a continuous distribution over possible predictions, is not straightforward. In a recent work, [8] suggested a definition based on credible intervals where if we take the *p* percentiles of each predicted distribution, the output should fall below them for exactly *p* percent of the data. Based on this definition, the authors further suggested a calibration evaluation metric and re-calibration method. While this seems very sensible and has the advantage of considering the entire distribution, we found serious flaws in this definition. The main problem arises from averaging over the entire dataset. We show, both empirically and analytically, that one can calibrate using this evaluation metric practically any output distribution, even one which is entirely uncorrelated with empirical uncertainty. We elaborate on this property of the evaluation method described in [8] in Section 2 and show empirical evidence in Section 4.

We propose a new, simple definition for calibration for regression, which is closer to the standard one for classification. Calibration for classification can be viewed as expecting the output for every single data point to correctly predict its error, in terms of misclassification probability. In a similar fashion, we define calibration for regression by simply replacing the misclassification probability with the mean square error. Based on this definition, we propose a new calibration evaluation metric similar to the expected calibration error (ECE) [9]. Finally, we propose a calibration method where we re-adjust the predicted uncertainty, in our case the outputted Gaussian variance, by minimizing the negative-log-likelihood (NLL) on a separate re-calibration set. We show good calibration results on a real-world dataset using a simple parametric model which scales the uncertainty by a constant factor. As opposed to [8], we show that our calibration metric does not claim that uncertainty that is uncorrelated with the real uncertainty is perfectly calibrated. To summarize, our main contributions are:

- Revealing the fundamental flaws in the current definition of calibrated regression uncertainty [8];
- A newly proposed definition of calibrated uncertainty in regression tasks, laying grounds for a new practical evaluation methodology;
- A simple scaling method, similar to temperature scaling for classification [6], that reduces calibration error in our experiments by the same amount as more complex methods.

### Related Work

While shallow neural networks are typically well-calibrated [10], modern, deep networks, albeit superior in accuracy, are no longer calibrated [6]. Uncertainty calibration for classification is a relatively studied field. Calibration plots or reliability diagrams provide a visual representation of uncertainty prediction calibration [10,11] by plotting an expected sample accuracy as a function of confidence. Confidence values are grouped into interval bins to allow computing the sample accuracy. A perfect model corresponds to the plot of the identity function. The expected calibration error (ECE) [9] summarizes the reliability diagram by averaging the error (gap between confidence and accuracy) in each bin, producing a single value measure of the calibration. Similarly, the maximum calibration error (MCE) [9] measures the maximal gap. Negative log likelihood (NLL) is a standard measure of a model’s fit to the data [12] but combines both the accuracy of the model and its uncertainty estimation in one measure. Based on these measures, several calibration methods were proposed, which transformed the network’s confidence output to one that will produce a calibrated prediction. Non-parametric transformations include histogram binning [13], Bayesian binning into quantiles [9], and isotonic regression [13] while parametric transformations include versions of Platt scaling [7] such as matrix scaling and temperature scaling [6]. In [6], it is demonstrated that the simple temperature scaling, consisting of a one scaling-parameter model, which multiplies the last layer logits, suffices to produce excellent calibration on many classification datasets.

In comparison with classification, the calibration of uncertainty prediction in regression has received little attention to date. As already described, [8] proposed a practical method for evaluation and calibration based on confidence intervals and isotonic regression. The proposed method is applied in the context of Bayesian neural networks. We present this method and analyzed it in the next section. In a recent work [14], the authors followed [8] the definition and method of calibration for regression, but used a standard deviation vs. MSE scatter plot, somewhat similar to our approach, as a sanity check. In concurrent work, ref. [15] proposed a calibration method that addresses the uniformity of [8] over the entire dataset. However, they do not address the inherent limitation in the calibration evaluation metric.

## 2. Confidence-Intervals Based Calibration

We then review the method for regression uncertainty calibration proposed in [8] which is based on confidence intervals, and highlight its shortcomings. We refer to this method in short as the “interval-based” calibration method. We start by introducing basic notations for uncertainty calibration used throughout the paper.

Notations. Let X,Y∼P be two random variables jointly distributed according to P and X×Y, their corresponding domains. A dataset ${\left\{\left(x_{t},y_{t}\right)\right\}}_{t=1}^{T}$ consists of i.i.d. samples of X,Y. A forecaster H:X→P(Y) outputs per example $x_{t}$ a distribution $p_{t}\equiv H\left(x_{t}\right)$ over the target space, where P(Y) is the set of all distributions over Y. In classification tasks, Y is discrete and $p_{t}$ is a multinomial distribution, and in regression tasks in which Y is a continuous domain, $p_{t}$ is usually a parametric probability density function, e.g., a Gaussian. For regression, we denote by $F_{t}:Y\to \left[0,1\right]$ the CDF corresponding to $p_{t}$.

According to [8], a forecaster in a regression setting *H* is calibrated if:

(1) $$\frac{\sum_{t=1}^{T}I\left\{y_{t}\leq F_{t}^{-1}\left(p\right)\right\}}{T}\overset{T\to \infty }{\to }p,\forall p\in \left[0,1\right]$$

Intuitively, this means that the $y_{t}$ is smaller than $F_{t}^{-1}\left(p\right)$ with probability *p*, or that the predicted CDF matches the empirical one as the dataset size goes to infinity. This is equivalent to

(2) $$P_{X,Y}\left(Y\leq {\left[F(X)\right]}^{-1}\left(p\right)\right)=p,\forall p\in \left[0,1\right]$$

where F(X) represents the CDF corresponding to H(X). This notion is translated by [8] to a practical evaluation and calibration methodology. A re-calibration dataset $S={\left\{\left(x_{t},y_{t}\right)\right\}}_{t=1}^{T}$ is used to compute the empirical CDF value for each predicted CDF value $p\in F_{t}\left(y_{t}\right)$:

(3) $$\hat{P}\left(p\right)=\frac{\left|\{y_{t}|F_{t}\left(y_{t}\right)\leq p,t=1\ldots T\}\right|}{T}$$

The calibration consists of fitting a regression function *R* (i.e., isotonic regression), to the set of points ${\left\{\left(p,\hat{P}\left(p\right)\right)\right\}}_{t=1}^{T}$. For diagnosis, the authors suggested a calibration plot of *p* versus $\hat{P}\left(p\right)$.

We start by intuitively explaining the basic limitation of this methodology. From Equation (3), $\hat{P}$ is non-decreasing and therefore isotonic regression finds a perfect fit. Therefore, the modified CDF $R\circ F_{t}$ will satisfy $\hat{P}\left(p\right)=p$ on the re-calibration set, and the new forecaster is calibrated up to sampling error. This means that perfect calibration is always possible, even for output CDFs which are statistically independent of the actual empirical uncertainty. We note that this might be acceptable when the uncertainty prediction degenerates, e.g., all output distributions are Gaussian with the same variance, but this is not the case here. We also note that the issue is with the calibration definition—not the re-calibration—as we show by following the analytic example.

We then present a concise analytic example in which the output distribution and the ground truth distribution are independent, yet fully calibrated according to Equation (2). Consider the case where the target has a normal distribution $y_{t}∼N\left(0,1\right)$ and the network output $H(x_{t})$ has a Cauchy distribution with a zero location parameter and random scale parameter $\gamma_{t}$ independent of $x_{t}$ and $y_{t}$, defined as:

(4) $$\begin{array}{cc} z_{t}∼ & N(0,1)\\ \gamma_{t}= & |z_{t}|\\ H(x_{t})= & Cauchy(0,\gamma_{t}) \end{array}$$

Following a known equality for Cauchy distributions, the CDF output of the network $F_{t}\left(y\right)=F\left(\frac{y}{\gamma_{t}}\right)$, where *F* is the CDF of a Cauchy distribution with zero location and 1 scale parameters. First, we note that $\frac{y_{t}}{\gamma_{t}}$ and $\frac{y_{t}}{z_{t}}$, i.e., with and without the absolute value, have the same distribution due to symmetry. We then recall the well-known fact that the ratio of two independent normal random variables is distributed as Cauchy with zero location and 1 scale parameters (i.e., $\frac{y_{t}}{z_{t}}∼Cauchy\left(0,1\right)$). This means that the probability that $F_{t}\left(y_{t}\right)\equiv F\left(\frac{y_{t}}{\gamma_{t}}\right)\leq p$ is exactly *p* (recall that *F* is a Cauchy(0,1) CDF). In other words, the prediction is perfectly calibrated according to the definition in Equation (2), even though the scale parameter was random and independent of the distribution of $y_{t}$.

While the Cauchy distribution is a bit unusual due to the lack of mean and variance, the example does not depend on it and it was chosen for its simplicity of exposition. It is possible to prove the existence of a distribution whose product of two independent samples is Gaussian [16] and replace the Cauchy with a Gaussian, but it is an implicit construction and not a familiar distribution.

## 3. Our Method

We present a new definition for calibration for regression, as well as several evaluation measures and a reliability diagram for calibration diagnosis, analogous to the ones used for classification [6]. The basic idea is that, for each value of uncertainty measured through standard deviation σ, the expected mistake, measured in mean square error (MSE), matches the predicted error $\sigma^{2}$. This is similar to the classification with MSE replacing the role of the misclassification error. More formally, if μ(x) and $\sigma {\left(x\right)}^{2}$ are the predicted mean and variance, respectively, then we consider a regressor to be well-calibrated if:

(5) $$\forall \sigma :\;\;E_{x,y}\left[{\left(\mu \left(x\right)-y\right)}^{2}{|\sigma \left(x\right)}^{2}=\sigma^{2}\right]=\sigma^{2}.$$

In contrast to [8], this does not average over points with different values of $\sigma^{2}$ at least in the definition; for practical measures, some binning is needed. In addition, compared to [15], it only looks at how well the MSE is predicted, separately from the quality of the prediction themselves, and similarly to classification where calibration and accuracy are disconnected. We claim that this captures the desired meaning of calibration, i.e., for each individual example, one can correctly predict the expected mistake.

Since we can expect each exact value of $\sigma^{2}$ in our dataset to appear exactly once, we empirically evaluate Equation (5) using binning, same we do for classification. Formally, let $\sigma_{t}$ be the standard deviation of the predicted output PDF $p_{t}$ and assume that, without loss of generality, the examples are ordered by increasing values of $\sigma_{t}$. We also assume for notation simplicity that the number of bins, *N*, divides the number of examples, *T*. We divide the indices of the examples into *N* bins, ${\left\{B_{j}\right\}}_{j=1}^{N}$, such that: $B_{j}=\left\{\left(j-1\right)\cdot \frac{T}{N}+1,\ldots ,j\cdot \frac{T}{N}\right\}$. Each resulting bin therefore represents an interval in the standard deviation axis: $\left[\min_{t\in B_{j}}\left\{\sigma_{t}\right\},\max_{t\in B_{j}}\left\{\sigma_{t}\right\}\right]$. The intervals are non-overlapping and their boundary values are increasing.

To evaluate how calibrated the forecaster is, we compare per bin *j* two quantities as follows. The root of the mean variance:

(6) $$RMV\left(j\right)=\sqrt{\frac{1}{|B_{j}|}\sum_{t\in B_{j}}\sigma_{t}^{2}}$$

Furthermore, the empirical root mean square error:

(7) $$RMSE\left(j\right)=\sqrt{\frac{1}{|B_{j}|}\sum_{t\in B_{j}}{\left(y_{t}-\hat{y_{t}}\right)}^{2}}$$

where ${\hat{y}}_{t}$ is the mean of the predicted PDF ($p_{t}$).

For diagnosis, we propose a reliability diagram which plots the RMSE as a function of the RMV. The idea is that, for a calibrated forecaster per bin, the RMV and the observed RMSE should be approximately equal, and hence, the plot should be close to the identity function. Apart from this diagnosis tool which, as we will show, is valuable for assessing calibration, we propose additional scores for evaluation.

Expected Normalized Calibration Error (ENCE). For summarizing the error in the calibration we propose the following measure:

(8) $$ENCE=\frac{1}{N}\sum_{j=1}^{N}\frac{\left|RMV(j)-RMSE(j)\right|}{RMV(j)}$$

This score averages the calibration error in each bin, normalized by the bin’s mean predicted variance, since for a larger variance, we expect naturally larger errors. This measure is analogous to the expected calibration error (ECE) [9] used in classification:

(9) $$ECE=p\left(i\right)∥o_{i}-e_{i}|$$

Here, the examples are split into *K* equal length interval bins according to the prediction confidence. $o_{i}$ is the true fraction of positive instances in bin *i*, $e_{i}$ is the mean of the post-calibrated probabilities for the instances in bin *i*, and P(i) is the empirical probability (fraction) of all instances that fall into bin *i*.

STDs Coefficient of variation ($C_{V}$). In addition to the calibration error, we would like to measure the dispersion of the predicted uncertainties. If, for example, the forecaster predicts a single homogeneous uncertainty measure for each example, which matches the empirical uncertainty of the predictor for the entire population, then the ENCE would be zero, but the uncertainty estimation per example would be uninformative. Therefore, we complemented the ENCE measure with the coefficient of variation ($c_{v}$) for the predicted STDs which measures their dispersion:

(10) $$c_{v}=\frac{\sqrt{\frac{\sum_{t=1}^{T}{\left(\sigma_{t}-\mu_{\sigma }\right)}^{2}}{T-1}}}{\mu_{\sigma }}$$

where $\mu_{\sigma }=\frac{1}{T}\sum_{t=1}^{T}\sigma_{t}$. Ideally, the $c_{v}$ should be high to indicate a dispersed uncertainty estimation over the dataset. We propose using the ENCE as the primary calibration measure and the $c_{v}$ as a secondary diagnostic tool.

### 3.1. Calibration

To understand the need for calibration, let us start by considering a trained neural network for regression, which has very a low mean squared error (MSE) on the train data. We now add a separate branch that predicts uncertainty as standard deviation, which together with the original network output interpreted as the mean, defines a Gaussian distribution per example. In this case, the NLL loss on the train data can be minimized by lowering the standard deviation of the predictions, without changing the MSE on train or test data. On test data, however, MSE will be naturally higher. Since the predicted STDs remain low on test examples, this will result in higher NLL and ENCE values for the test data. This type of miscalibration is defined as over-confidence, but opposite or mixed cases can occur depending on how the model is trained.

Negative log-likelihood. NLL is a standard measure for a probabilistic model’s quality [12]. When training the network to output classification confidence or a regression distribution, it is commonly used as the objective function to minimize. It is defined as:

(11) $$NLL=-\sum_{t=1}^{T}log\left(\left[H\left(x_{t}\right)\right]\left(y_{t}\right)\right)$$

We propose using the NLL on the re-calibration set as our objective for calibration, and the reliability diagram, together with its summary measures (ENCE, $c_{v}$) for diagnosis of the calibration. In the most general setting, a calibration function maps predicted PDFs to calibrated PDFs: R(Θ):P(Y)→P(Y) where θ is the set of parameters defining the mapping.

Optimizing calibration over the re-calibration set is obtained by finding θ yielding minimal NLL:

(12) $$\arg\min_{\theta }\left(-\sum_{t=1}^{T}log\left(\left[R(p_{t};\Theta )\right]\left(y_{t}\right)\right)\right).$$

To ensure the calibration generalization, the diagnosis should be made on a separate validation set. Multiple choices exist for the family of functions that *R* belongs to. We propose using *STD Scaling*, (in analogy to temperature scaling [6]), which essentially multiplies the STD of each predicted distribution by a constant scaling factor *s*. If the predicted PDF is that of a Gaussian distribution, $N(\mu ,\sigma^{2})$, then the re-calibrated PDF is $N(\mu ,{\left(s\cdot \sigma \right)}^{2})$. Hence, in this case, the calibration objective (Equation (12)) is:

(13) $$\begin{array}{c} \arg\min_{s}\left(\frac{T}{2}log\left(s\right)-\sum_{t=1}^{T}\frac{{\left(y_{t}-\mu_{t}\right)}^{2}}{2s^{2}\sigma_{t}^{2}}\right) \end{array}$$

If the original predictions are overconfident, as common in neural networks, then the calibration should set s>1. This is analogous to temperature scaling in classification: a single multiplicative parameter is tuned to fix the over- or under-confidence of the model, and it does not modify the model’s final prediction since $\mu_{t}$ remains unchanged.

More complex calibration methods. Histogram binning and Isotonic Regression applied to the STDs can be also used as calibration methods. We chose STD scaling since: (a) it is less prone to overfit the validation set; (b) it does not enforce minimal and maximal STD values; (c) it is easy to implement; and (d) empirically, it produced good calibration results, on par with the much more complex percentile-based isotonic regression of [8].

## 4. Experimental Results

We then show the empirical results of our approach on two tasks: a controlled synthetic regression problem and object detection bounding box regression. We examine the effect of outputting trained and random uncertainty on the calibration process. In all training and optimization stages, we use an SGD optimizer with a learning rate of 0.001 and a momentum of 0.9.

We note that, since the calibration in [8] works by directly changing the CDF, we need to extract the variance from the modified CDF. To do that, we use the formula

(14) $$\sigma^{2}=2\int_{0}^{\infty }u\left(1-F\left(u\right)\right)du-{\left(\int_{0}^{\infty }\left(1-F\left(u\right)\right)du\right)}^{2}$$

We numerically calculate the integral in Equation (14) using Romberg’s integration method.

### 4.1. Synthetic Regression Problem

Experimenting with a synthetic regression problem enables us to control the target distribution *Y* and validate our method. We randomly generate *T* = 50,000 input samples ${\left\{x_{t},y_{t}\right\}}_{t=1}^{T}$. We sample $x_{t}$ from X∼Uniform[0.1,1] and $y_{t}$ from $Y∼N(x_{t},x_{t}^{2})$. This way, the target standard deviation of sample $x_{t}$ is $x_{t}$. We train a fully connected network with four layers and a ReLU activation function on the generated training set using the $L_{1}$ loss function. In this random uncertainty experiment, per example, the standard deviation representing the uncertainty is randomly drawn from Uniform[1,10]. We then re-calibrate as described in Section 3.1 on a separate re-calibration set consisting of 6000 samples.

**Figure 1:**
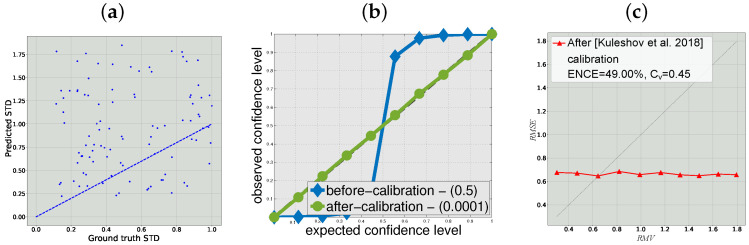
Results for the synthetic regression problem with random uncertainty estimation: (**a**) real vs. predicted STDs after confidence interval calibration [8]; (**b**) confidence intervals method evaluation of calibrated random uncertainty; and (**c**) reliability diagram using our evaluation method after [8] calibration. Grey dashed line indicates the ideal calibration.

As one can see in Figure 1b the, confidence interval method [8] can almost perfectly calibrate the random independent uncertainty estimation according to their definition, as the expected and observed confidence level match and we obtain the desired identity curve. This phenomenon is extremely undesirable for safety critical applications where falsely relying on uninformative uncertainty can lead to severe consequences. In Figure 1a, we show the predicted STD vs. real STD after this calibration showing that the predictions that are perfectly calibrated according to the interval definition are indeed uncorrelated with the actual uncertainty. In contrast, one can see in Figure 1c that the these results are clearly un-calibrated by our definition and metric.

### 4.2. Bounding Box Regression for Object Detection

An object detector outputs per input image a set of bounding boxes, each commonly defined by five outputs: classification confidence and four positional outputs $\left(t_{x},t_{y},t_{w},t_{h}\right)$ representing its (x,y) position, width and height. As our base architecture, we use the R-FCN detector [17] with a ResNet-101 backbone [18]. The R-FCN regression branch outputs per region candidate a 4D vector that parameterizes the bounding box as $t_{b}=\left(t_{x},t_{y},t_{w},t_{h}\right)$ following the accepted parameterization in [19]. We use these outputs in our experiments as four separate regression outputs. To this architecture, we add an uncertainty branch, identical in structure to the regression branch, which outputs a 4D vector $\left(u_{1},u_{2},u_{3},u_{4}\right)\equiv \left(log\left(\sigma_{x}^{2}\right),log\left(\sigma_{y}^{2}\right),log\left(\sigma_{w}^{2}\right),log\left(\sigma_{h}^{2}\right)\right)$, each representing the log variance of the Gaussian distributions of the corresponding output. As before, the original regression output represents the Gaussian mean (i.e., $\mu_{x}=t_{x}$). Thus, the network outputs a Gaussian distribution per regression task.

For training the network weights, we used the entire Common Objects in Context (COCO) dataset [20]. The COCO dataset contains over 300K images of everyday objects and humans. Among the COCO benchmarks, we train on the object detection task. The ground truth for this task consists of marked coordinates of object bounding boxes for 80 categories. For uncertainty calibration and validation, we use two separate subsets of the KITTI [21] object detection benchmark dataset. This benchmark contains 14 K images taken in road scenes with 80 K annotated objects belonging to seven road occupant classes (e.g., car, pedestrian). We perform a mapping from the relevant COCO classes to the KITTI classes. Training the uncertainty output on one dataset and performing calibration on a different one without changing the predictions reduces the risk of over-fitting and increases the calibration validity.

We initially train the network without the additional uncertainty branch as in [17], while the uncertainty branch weights are randomly initialized. We then train the uncertainty branch by minimizing the NLL loss (Equation (11)) on the training set, freezing all network weights but the uncertainty head for 1K training iterations with six images per iteration. Freezing the rest of the network ensures that the additional uncertainty estimation represents uncertainty on unseen data. The result of this stage is the network with predicted uncertainty. Finally, we train the NLL loss for 1K additional training iterations on the re-calibration set, to optimize the single scaling parameter *s* and obtain the calibrated uncertainty.

**Figure 2:**
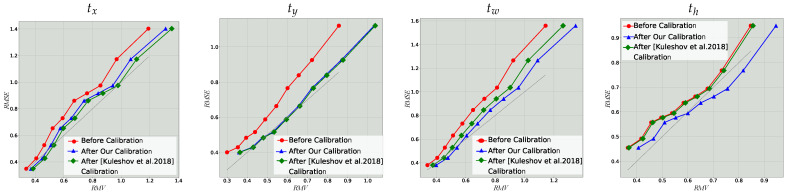
Reliability diagrams for bounding box regression on the KITTI validation set before and after calibration. Each plot compares the empirical RMSE and the root mean variance (RMV) in each bin. Grey dashed line indicates the ideal calibration line [8]. See Section 4.2 for details.

Figure 2 shows the resulting reliability diagrams before calibration (predicted uncertainty) and after (calibrated uncertainty) for all four positional outputs, on the validation set. For comparison, we also show the results are calibrated with the interval method [8]. As can be observed from the monotonously increasing curve before calibration, the output uncertainties are indeed correlated with the empirical ones. Additionally, since the curves are entirely above the ideal one, the predictions are over confident. Using the learned scaling factor *s*, which varies between 1.1 and 1.2, the ENCE is significantly reduced as shown in Table 1. The $c_{v}$ remains unchanged after calibration since it is invariant to the uniform scaling of the output STDs (Equation (10)).

In Table 1, we see that calibration with both our method and the method in [8] considerably improves the ENCE and have comparable performance. We first note that our calibration is much simpler, with only a scalar parameter compared to isotonic regression and does not need any numeric integration to calculate the mean and variance, unlike the calibration in [8]. Furthermore, we observe that while we showed that the definition of calibration in [8] and evaluation is flawed, the re-calibration algorithm that was derived from it still shows good results.

## 5. Conclusions

Calibration, and more generally uncertainty prediction, are critical parts of machine learning, especially in safety-critical applications. In this work, we exposed serious flaws in the current approach to define and evaluate calibration for regression problems. We proposed a new definition for calibration in regression problems and evaluation metrics. Based on our definition, we proposed a simple re-calibration method that showed significant improvement in real-world applications. Further research is required to test the generalization of our evaluation to multiple domain and tasks. In addition, it may be the case that, in other tasks, more complex calibration methods are required.

## Figures and Tables

**Table 1:** Evaluation of uncertainty calibration for the bounding box regression tasks on the KITTI validation dataset.

	Before Calibration		Calibrated (Ours)		Calibrated [8]	
	ENCE	$C_{v}$	ENCE	$C_{v}$	ENCE	$C_{v}$
$t_{x}$	16.5%	0.40	8.3%	0.40	**6.0%**	0.40
$t_{y}$	25.4%	0.33	4.7%	0.33	**3.9%**	0.33
$t_{w}$	24.4%	0.38	**8.4%**	0.38	12.6%	0.38
$t_{h}$	12.6%	0.26	**5.7%**	0.26	11.2%	0.26

## Data Availability

For training the network weights, we used the Common Objects in Context (COCO) dataset [20]. For uncertainty calibration and validation, we use the KITTI [21] object detection benchmark dataset.

## Abbreviations

The following abbreviations are used in this manuscript:

STD	Standard deviation
ECE	Expected calibration error
MCE	Minimum calibration error
NLL	Negative log likelihood
ENCE	Expected normalized calibration error
PDF	Probability density function
CDF	Cumulative distribution function
RMSE	Root mean squared error
EMV	Root mean variance
